# Effect of conditional deletion of cytoplasmic dynein heavy chain DYNC1H1 on postnatal photoreceptors

**DOI:** 10.1371/journal.pone.0248354

**Published:** 2021-03-11

**Authors:** Tiffanie M. Dahl, Michelle Reed, Cecilia D. Gerstner, Guoxin Ying, Wolfgang Baehr

**Affiliations:** 1 Department of Ophthalmology, University of Utah Health Science Center, Salt Lake City, Utah, United States of America; 2 Department of Neurobiology & Anatomy, University of Utah, Salt Lake City, Utah, United States of America; 3 Department of Biology, University of Utah, Salt Lake City, Utah, United States of America; National Eye Centre, UNITED STATES

## Abstract

Cytoplasmic dynein (dynein 1), a major retrograde motor of eukaryotic cells, is a 1.4 MDa protein complex consisting of a pair of heavy chains (DYNC1H1) and a set of heterodimeric noncatalytic accessory components termed intermediate, light intermediate and light chains. DYNC1H1 (4644 amino acids) is the dynein backbone encoded by a gene consisting of 77 exons. We generated a floxed *Dync1h1* allele that excises exons 24 and 25 and truncates DYNC1H1 during Six3Cre-induced homologous recombination. Truncation results in loss of the motor and microtubule-binding domain. *Dync1h1*^F/F^;Six3Cre photoreceptors degenerated rapidly within two postnatal weeks. In the postnatal day 6 (P6) *Dync1h1*^F/F^;Six3Cre central retina, outer and inner nuclear layers were severely disorganized and lacked a recognizable outer plexiform layer (OPL). Although the gene was effectively silenced by P6, DYNC1H1 remnants persisted and aggregated together with rhodopsin, PDE6 and centrin-2-positive centrosomes in the outer nuclear layer. As photoreceptor degeneration is delayed in the *Dync1h1*^F/F^;Six3Cre retina periphery, retinal lamination and outer segment elongation are in part preserved. DYNC1H1 strongly persisted in the inner plexiform layer (IPL) beyond P16 suggesting lack of clearance of the DYNC1H1 polypeptide. This persistence of DYNC1H1 allows horizontal, rod bipolar, amacrine and ganglion cells to survive past P12. The results show that cytoplasmic dynein is essential for retina lamination, nuclear positioning, vesicular trafficking of photoreceptor membrane proteins and inner/outer segment elaboration.

## Introduction

Mouse photoreceptor rod outer segments (OS) are continuously renewed every ten days [1, 2]. This high degree of turnover requires reliable delivery of large amounts of transmembrane (TM) protein to the OS following the conventional secretory pathway [3] (reviewed in [4]). Trafficking of vesicles destined for the OS occurs by transport along microtubules by the minus-end directed molecular motor, cytoplasmic dynein [5–7]. Dynein cargo may include mitochondria, membrane vesicles, lysosomes, phagosomes, nuclei and other organelles [8].

Dynein is a 1.4 MDa protein complex composed of a pair of force-generating heavy chains (DYNC1H1) and a set of heterodimeric noncatalytic accessory components termed intermediate, light intermediate and light chains. The dynein motor assembles around a ring of six AAA+ (ATPases Associated with various Activities) domains of the heavy chain. The microtubule (MT)-binding domain sits at the tip of a coiled-coiled stalk emerging from AAA4. To move along the MT track, the motor domain couples ATP hydrolysis with a mechanochemical cycle (reviewed in [9]). Noncatalytic subunits participate in complex assembly, stability, motor-cargo interactions and motor activity regulation [10]. Aside from cargo binding, activating adaptor proteins (effectors) also recruit dynactin, a 1 MDa complex consisting of 23 subunits. Dynactin functions as a dynein co-factor that enables dynein to move membrane vesicles along microtubules.

A *dync1h1* nonsense mutation (Y3102X) underlies the *cannonball (cnb)* phenotype in zebrafish [7]. The truncated protein lacks the carboxyterminal one-third of Dync1h1 including the stalk domain where microtubule binding occurs, as well as the fifth and sixth ATPase motor domains, yet *cnb* embryos survive until larval stages. Retinal photoreceptor neurons, however, exhibit defects in organelle positioning, post-Golgi vesicle trafficking and OS morphogenesis. GFP-tagged rhodopsin mislocalized in both the *cannonball* mutant and *dync1h1* morphant cells [7]. A mutation in the zebrafish *mikre oko* (mok) locus, which encodes the dynactin 1 subunit of the dynein complex, results in a severe displacement of the photoreceptor nucleus toward the synaptic terminus, but does not interfere with rhodopsin trafficking [11].

Germline deletions of mouse *Dync1h1* (truncation after exon 1) are lethal as embryos do not survive beyond E8.5 [12]. Three mouse mutants, ’legs at odd angles’ (*Loa*), ’Cramping 1’ (*Cra1*) and ‘Sprawling’ (*Swl*) display autosomal dominant mouse phenotypes that arose from either ENU mutagenesis or radiation generating missense mutations in the *Dync1h1* tail domain. *Cra1*/+ mice exhibit early onset stable behavioral deficits, including abnormal hind limb posturing and decreased grip strength [13]. *Loa*/+ mice exhibit defects in retrograde axonal transport [14] and neuronal migration [15]. *Loa* and *Cra1* mutations exhibit remarkable similarities to specific features of human pathology for amyotrophic lateral sclerosis (ALS) [16]; *Loa*, *Cra1* and *Swl* retina phenotypes have not been assessed. *Dync1h1* mutations in human have been associated with spinal muscular atrophy and Charcot-Marie-Tooth Disease [17–20].

We generated conditional knockouts of *Dync1h1* in mouse using Six3Cre that initiates ablation of DYNC1H1 after embryonic day 9 during prenatal development of retina and RPE progenitors. Deletion of DYNC1H1 has severe consequences for retinogenesis. Initial segregation of the ONL/INL, observed at P6, is absent at P8 when mutant photoreceptors appear to lack inner and outer segments. Foci of immunoreactivity positive for rhodopsin, S-opsin or rod PDE6 were sequestered within the ONL or at the ONL distal edge. Mutant cones survived longer than rods. DYNC1H1 was detectable in the IPL and GCL through P16, although Cre-mediated excision of exons 24 and 25 of *Dync1h1* had occurred throughout most of the retina by P6, suggesting unsuccessful clearance of the DYNC1H1 polypeptide.

## Results

### Generation of the conditional knockouts

**Fig 1:**
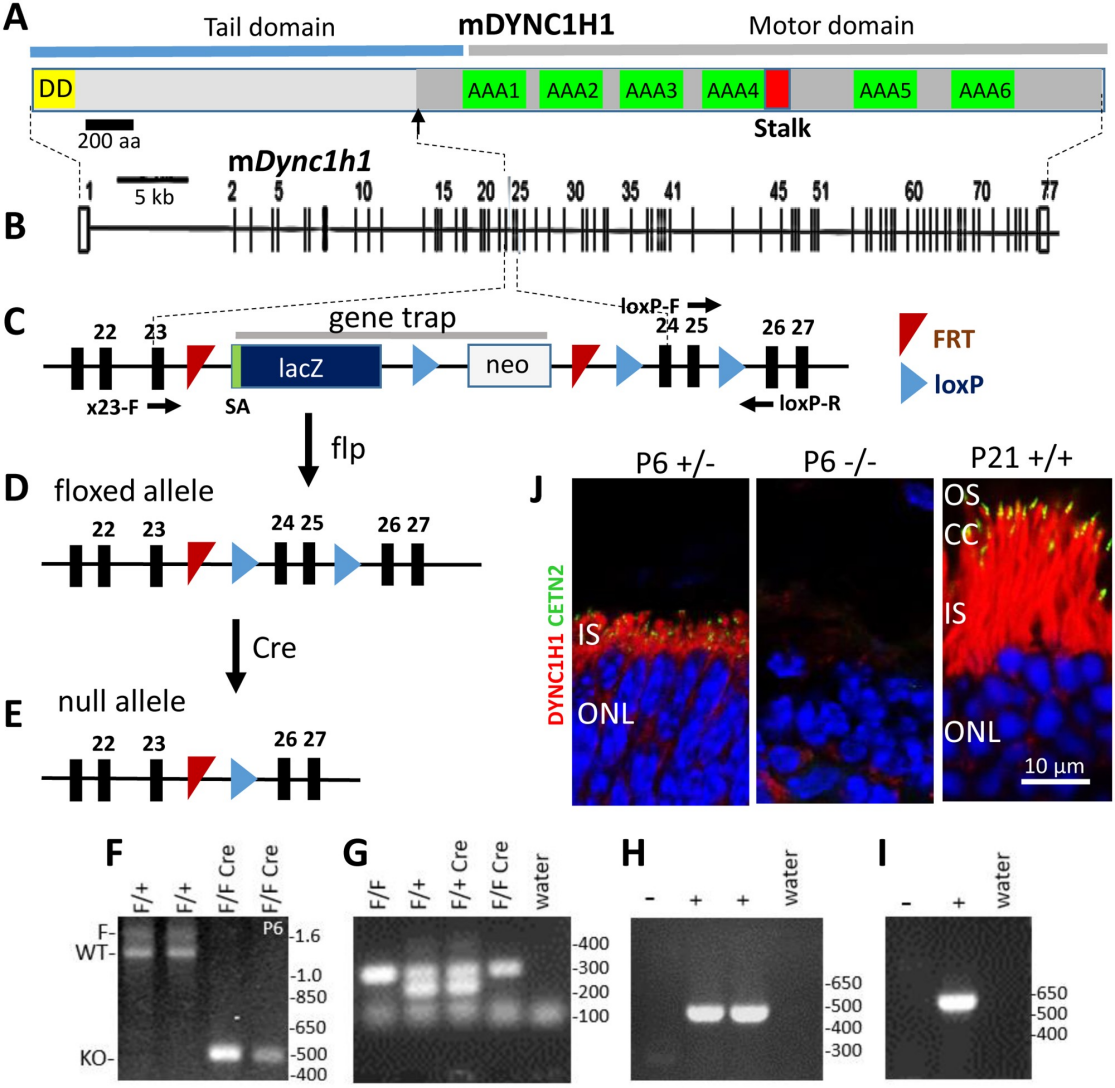
Generation of *Dync1h1* conditional knockouts. **A**, schematic of mouse dynein heavy chain (DYNC1H1, gene symbol *Dync1h1*) consisting of an N-terminal tail and a C-terminal motor domain. The motor is built around a ring of 6 AAA+ (ATPases Associated with various Activities) units of the heavy chain. The MT-binding domain sits at the tip of a coiled-coiled stalk (red) emerging from the C-terminal end of AAA4. **B**, the mouse *Dync1h1* gene with 77 exons. **C**, enlargement of the gene trap in intron 23 consisting of a splice acceptor site (SA), a β-galactosidase reporter (lacZ), and a neo cassette flanked by loxP sites. A third loxP site is present in intron 25. Horizontal black arrows approximate positions of genotyping primers. **D**, floxed allele. **E**, null allele. **F,** PCR genotyping with primers exon 23-F (X23-F) and loxP-R confirming loss of exons 24/25. **G**, presence of loxP by PCR with loxP-F and loxP-R primers. **H**, presence of Six3Cre by PCR with Six3Cre-F and Six3Cre-R. **I**, presence of EGFP-CETN2 with CETN2-F and CETN2-R primers. **J**, DYNC1H1 immunolocalization (red) in P6 *Dync1h1*^F/+^;Six3Cre;Egfp-Cetn2 photoreceptors (left panel), P6 *Dync1h1*^F/F^;Six3Cre photoreceptors (middle panel) and P21 *Dync1h1*^+/+^;Egfp-Cetn2 photoreceptors (right panel). EGFP-CETN2 (green) identifies locations of centrioles and connecting cilia (CC). DAPI identifies ONL nuclei. Note DYNC1H1 is absent in the P6 *Dync1h1*^F/F^;Six3Cre IS. OS, outer segment; IS, inner segment; ONL, outer nuclear layer.

Mouse DYNC1H1 is a very large dimeric protein (4644 amino acids) which forms the backbone of cytoplasmic dynein. It features an N-terminal tail domain with binding sites for other regulatory components of the dynein complex and docking sites for cargoes, including adaptor proteins [21] (Fig 1A). DYNC1H1 folds into a motor domain comprising six ATPase domains (AAA1-6) and a microtubule binding stalk region at its C-terminal region. The mouse gene encoding DYNC1H1 consists of 77 exons (Fig 1B) spanning 65 kb of genomic DNA. To enable conditional knockouts, we acquired a cell line [22] in which a gene trap flanked by loxP and FRT sites was placed in intron 23 and a third loxP site was placed in intron 25 (Fig 1C). A floxed *Dync1h1* allele (*Dync1h1*^F^) (Fig 1D) is generated following FRT-FLP recombination with Flp-recombinase. Deletion of DYNC1H1 in retina was achieved by mating with Six3Cre transgenic mice [23] expressing Cre recombinase at embryonic day 9 (E9) to yield *Dync1h1*^F/F^;Six3Cre knockouts (Fig 1E); deletion of exons 24 and 25 truncates *Dync1h1* after exon 23 as exon 26 is out-of-frame. The truncation point is located prior to the motor domain (Fig 1A, arrow), and the truncated protein is likely nonfunctional since the motor domain and MT binding stalk are lost. Genotyping (Fig 1F–1I) confirmed loss of exons 24 and 25 at P6 (F), presence of loxP (G), (*Dync1h1*^F/F^ vs. *Dync1h1*^F/+^), presence of Six3Cre (H), and presence of EGFP-CETN2 (I) (for details see Methods). DYNC1H1 immunolocalizes prominently in the P21 photoreceptor inner segment (IS) (Fig 1J, right panel). At P6, DYNC1H1 is undetectable in the knockout IS (Fig 1J, middle panel). Breeding to generate *Dync1h1*^F/F^;Six3Cre knockouts occurred normally and with average litter sizes. Phenotypically we observed that *Dync1h1*^F/F^;Six3Cre mice have smaller eyes but the extent of microphthalmia varies, even between two eyes of one animal.

### Disrupted ONL/INL lamination of *Dync1h1*^F/F^;Six3Cre retina

**Fig 2:**
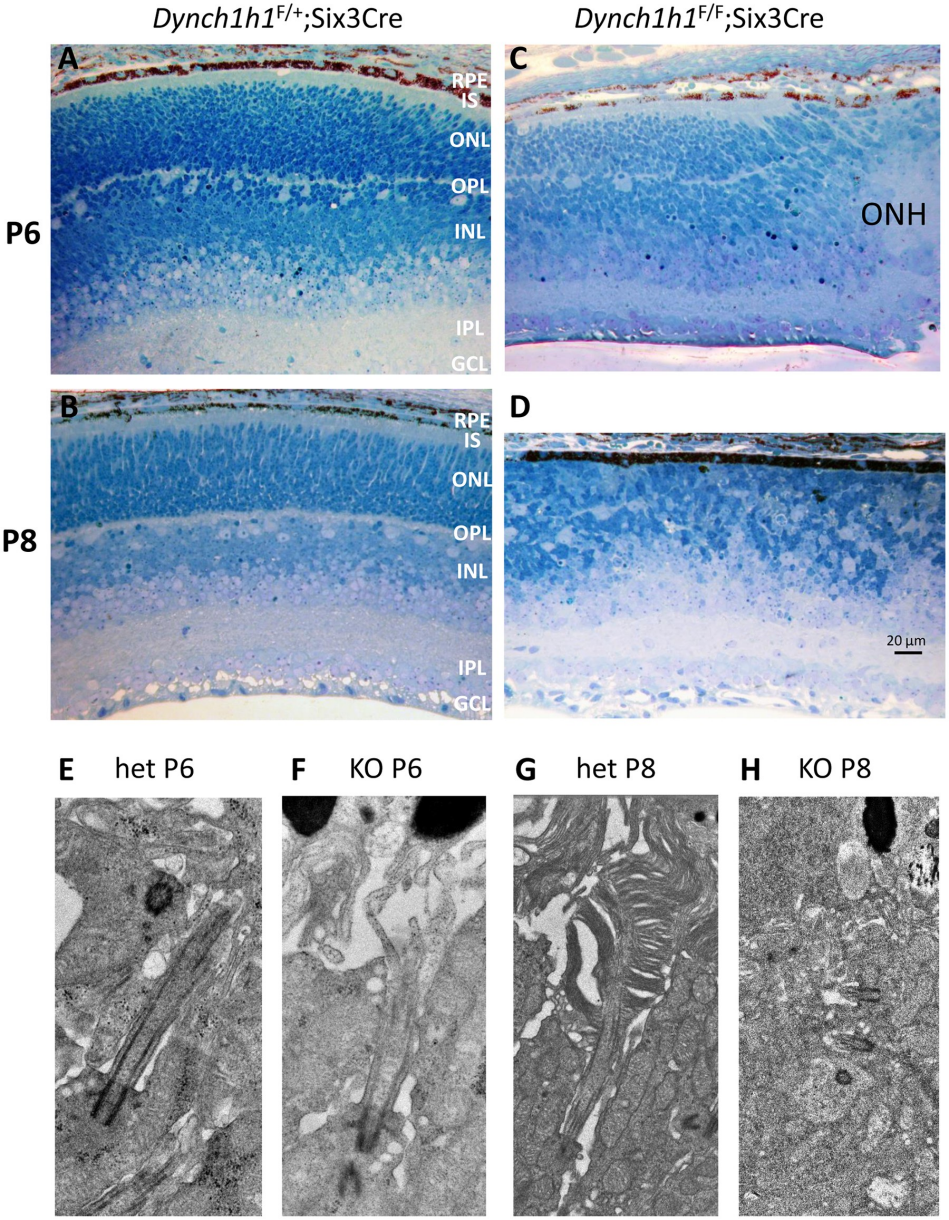
Defective *Dync1h1*^F/F^;Six3Cre retina lamination and impaired ciliogenesis. **A-D**, *Dync1h1*^F/+^;Six3Cre (A, B) and *Dync1h1*^F/F^;Six3Cre plastic sections (C, D) of littermate central retina near the optic nerve head (ONH) at P6 (A, C) and P8 (B, D). Sections stained with toluidine blue-Azure II (Richardson’s stain) to demonstrate retina layers. RPE, retinal pigmented epithelium; IS, inner segment; ONL, outer nuclear layer; OPL, outer plexiform layer; INL, inner nuclear layer; IPL, inner plexiform layer; GCL, ganglion cell layer. Scale bar, 20 μm. **E-H,** representative TEM images of connecting cilia emanating from heterozygous control (E, G) and *Dync1h1*^F/F^;Six3Cre basal bodies (F, H) at P6 and P8. Note absence of CC at P8 at *Dync1h1*^F/F^;Six3Cre basal bodies.

Six3 (*sine oculis*-related homeobox 3), a transcription factor expressed in retina, RPE and brain during embryonic development [23], is required for maintenance of multipotent retinal progenitors [24]. The effect of Six3Cre expression in *Dync1h1*^F/+^;Six3Cre and *Dync1h1*^F/F^;Six3Cre retinas was examined in transverse full-retina plastic sections at P6 and P8 (Fig 2A–2D). In *Dync1h1*^F/+^;Six3Cre control sections, the retina nuclear layers are well established at each time point (Fig 2A and 2B). In *Dync1h1*^F/F^;Six3Cre sections next to the optical nerve head (ONH), the outer plexiform layer (OPL) and IS can be distinguished in part at P6, but both are absent at P8 (Fig 2C and 2D). The RPE appears heavily pigmented, and the merged ONL/INL is reduced in thickness while the IPL appears minimally affected.

We investigated whether connecting cilia (CC), equivalent to transition zones of primary cilia, are formed at P6 when ciliogenesis begins. Transmission Electron Microscopy (TEM) images reveal rare examples where basal bodies docked to the IS membrane generating a CC (Fig 2E and 2F). At P8, control photoreceptors begin to form an outer segment (Fig 2G) whereas *Dync1h1*^F/F^;Six3Cre photoreceptors degenerate and discontinue CC and OS formation (Fig 2H).

### Absence of outer segments and functional OPL in DYNC1H1-depleted photoreceptors

**Fig 3:**
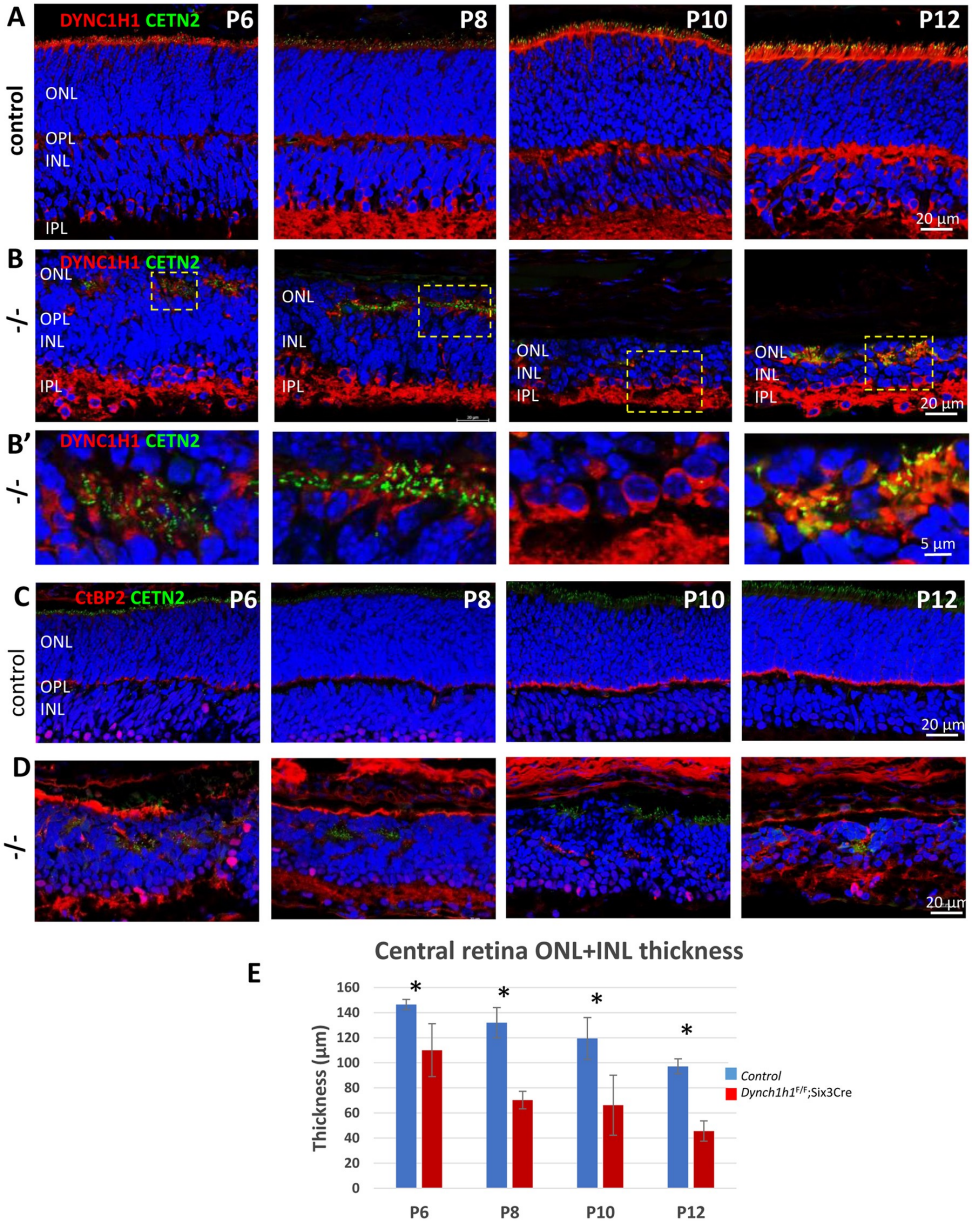
Disorganized nuclear layers in the absence of DYNC1H1. Row **A,** representative control Egfp-Cetn2 retina cryosections at P6-P12 probed with anti-DYNC1H1 (red). DAPI-labeled ONL and INL are clearly separated by the OPL at P6, DYNC1H1 is expressed in the IS and distal INL. DYNC1H1 is also present in the OPL at P8 and later. Row **B**, representative *Dync1h1*^F/F^;Six3Cre;Egfp-Cetn2 sections at P6-P12. At P6, the outer nuclear layer is severely disorganized; ONL and INL cannot easily be distinguished (no clear OLM or OPL). At P8 and later, the ‘merged’ nuclear layer shrinks successively. Note persistent expression of DYNC1H1 in the IPL. Row **B’**, enlarged row B regions showing structures with accumulation of EGFP-CETN2 and traces of DYNC1H1. Rows **C, D**, control (row C) and knockout sections (row D) probed with anti-CtBP2/Ribeye at P6-P12. CtBP2/Ribeye localizes specifically to rod ribbon synapses and cone pedicles of control sections. CtBP2/Ribeye accumulates at nuclear layer borders and becomes progressively undetectable in synaptic termini of knockout sections. **E**, quantitative analysis of combined (ONL and INL) nuclear layer thickness from P6 to P12. Student’s t-test, * indicates p<0.05.

DYNC1H1 is expressed prominently in the photoreceptor IS of control retinas with traces of DYNC1H1 in ONL somata, OPL and IPL (Fig 3A). In *Dync1h1*^F/F^;Six3Cre cryosections (Fig 3, row **B**), OS are unrecognizable and DYNC1H1 is absent at locations where IS should have formed. DYNC1H1 apparently persists in the IPL (Fig 3, row **B**) although exons 24 and 25 of *Dync1h1* were excised by P6 (Fig 1F) suggesting that DYNC1H1 could not be cleared by the ubiquitin proteasome or autophagy-lysosome pathway [25].

In the P6 *Dync1h1*^F/F^;Six3Cre central retina, the OPL is fragmented, and a border between ONL and INL is often unrecognizable, suggesting that bipolar cell dendrites do not develop well (Fig 3, rows **B**, **D**). The combined (ONL and INL) nuclear layer thickness is reduced by 20% at P6 (Fig 3E) suggesting reduced neurogenesis or premature cell death. Centrosomes, identified by EGFP-CETN2, fail to extend connecting cilia and aggregate with traces of DYNC1H1 in foci (Fig 3, row **B’**, P6 and P8). The merged ONL/INL progressively shrinks from P6 to P12 and reveals 2–5 nuclear rows by P12 (Fig 3E). We probed for presence of CtBP2/Ribeye, a rod and cone synaptic marker delineating the OPL in controls (Fig 3, row **C**) [26], and observed that mutant photoreceptor synaptic termini are devoid of CtBP2/Ribeye (Fig 3, row **D**) suggesting absence of functional ribbon synapses. The results support a role for dynein in postnatal nuclear migration, lamination and ONL/INL architecture.

### Dynein organizes membrane protein trafficking and photoreceptor polarization

**Fig 4:**
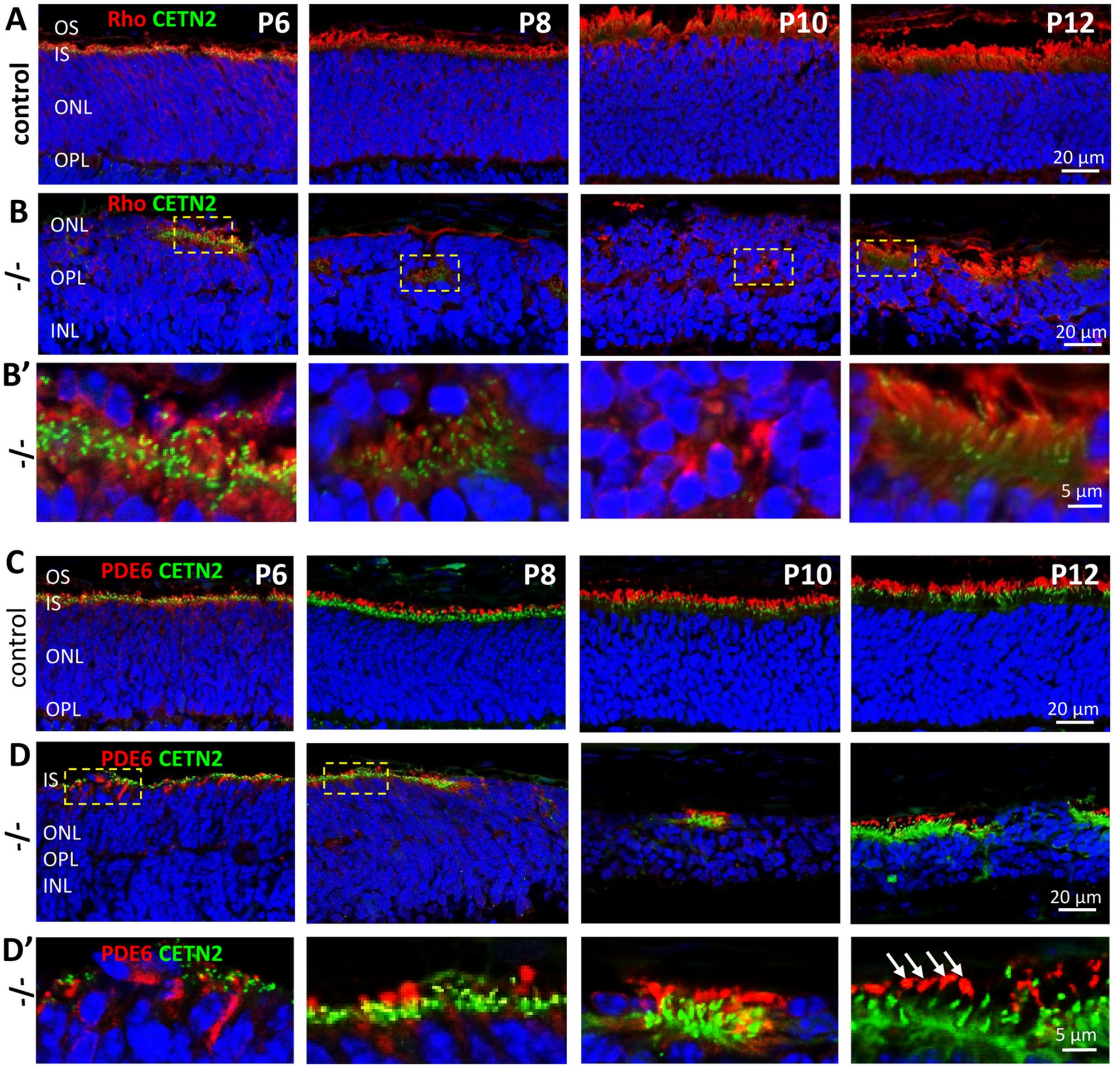
DYNC1H1 retina deletion affects rhodopsin, PDE6. Row **A, B,** cryosections of control (row A) and *Dync1h1*^F/F^;Six3Cre;Egfp-Cetn2 (row B) retinas at P6-P12 probed with anti-rhodopsin (mAb 4D2, red). In row B, nuclei of ONL and INL intermingle and synaptic termini become difficult to delineate. At P8 and later, rhodopsin aggregates with EGFP-CETN2-positive centrioles (green) in the rod distal IS. Row **B’**, enlargements of row B sections containing rhodopsin and centrioles, as indicated. Rows **C, D**, cryosections of control (row C) and knockout *Dync1h1*^F/F^;Six3Cre;Egfp-Cetn2 (row D) retinas probed with MOE (red). PDE6, a peripherally attached membrane protein, transports to the OS independent of dynein. In row **D,** PDE6, expressed at low levels at P6-P10, aggregates with centrioles in the nuclear layer. Row **D**’, enlarged regions of row D, as indicated. Note the miniscule PDE6-positive profiles presumed to be cone OS.

Developmental processes defining photoreceptor polarity, i.e., synaptogenesis versus OS elaboration, are key to maturation of sensory neurons. We examined the fates of two prominent OS proteins, rhodopsin and cGMP phosphodiesterase (PDE6), in the dynein knockout environment relative to controls. In P6 control retina, rhodopsin is located in the distal IS near the basal body; rhodopsin localizes to the ROS exclusively at P8 and later (Fig 4, row **A**). In the *Dync1h1*^F/F^;Six3Cre central retina at P6 and P8 (Fig 4, row **B**), ROS fail to form and rhodopsin is deposited together with centrioles in disorganized pockets within the nuclear layer, likely incorporated into ER membranes (Fig 4, row **B’**). At P10 and P12, rhodopsin accumulates around nuclei and in sloughed membrane at the sclerad edge of the ONL. At P16, the nuclear layer consists of one row of nuclei presumably consisting of surviving mutant cones (results not shown).

Rod and cone PDE6, key enzymes of the phototransduction cascade, are heterotetrameric soluble proteins that are membrane-anchored by isoprenylation. Both are synthesized in the cytosol, dock to the ER for posttranslational processing (isoprenylation) and traffic to the OS in a complex with PDE6D by diffusion, independent of dynein [27]. Using anti-PDE6 antibody (MOE) that recognizes both rod and cone PDE6 [28], we show that PDE6 localizes exclusively to OS of control cryosections (Fig 4, row **C**). Iin P6 mutant sections, PDE6 accumulates together with centrosomes in a cytosolic space or in foci at the sclerad edge of the ONL (Fig 4, row **B**). At P8 and later, PDE6 accumulates in immunoreactive structures distal to the basal body (see Fig 4, row **D’** at P12, white arrows). We interpret the MOE-positive structures to be most likely diminutive remnants of rod or cone OS (lacking visual pigment) as observed in germline rhodopsin knockouts [29, 30].

### Inner retina cells survive longer than rods

**Fig 5:**
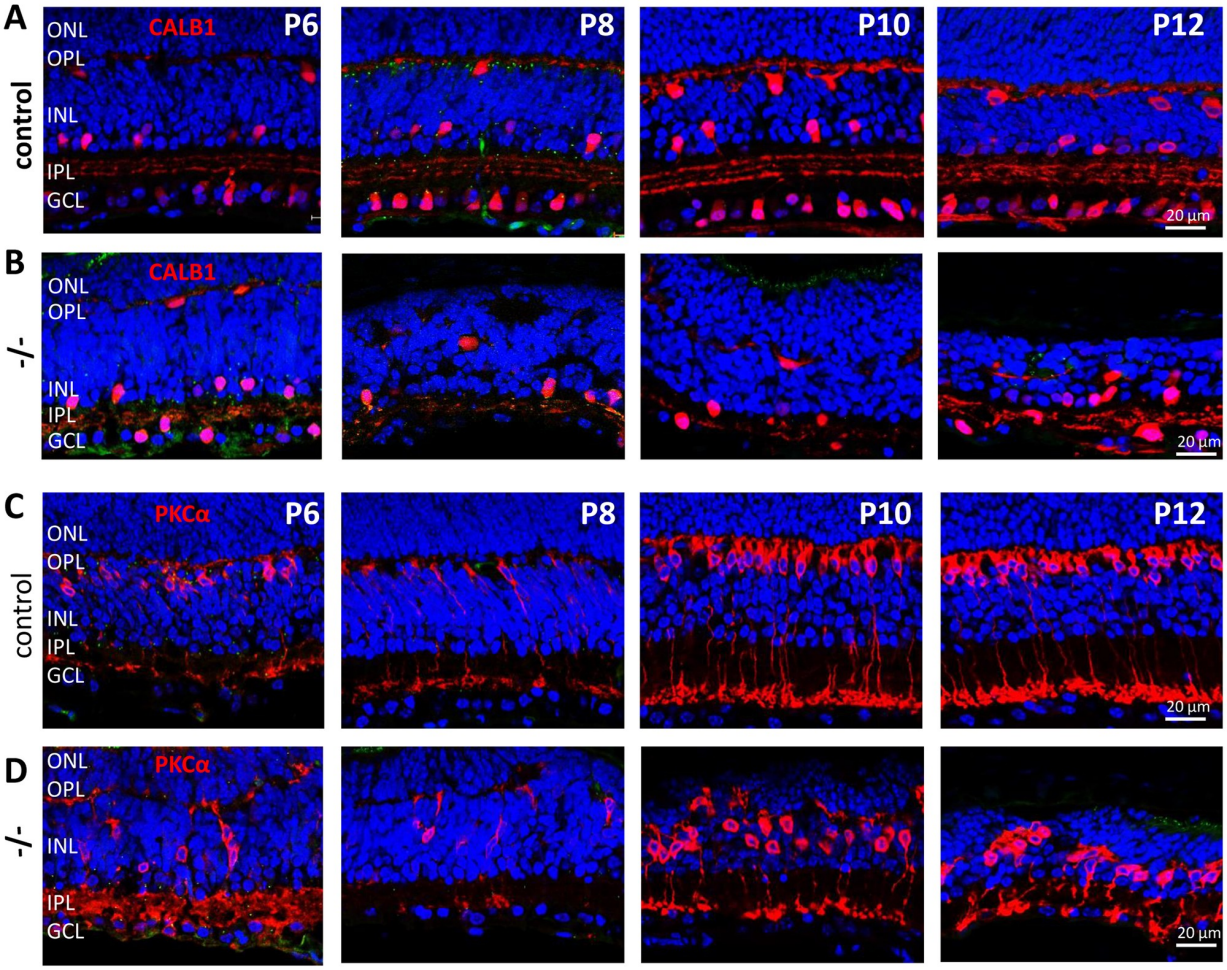
Inner retinal neurons in *Dync1h1*^F/F^;Six3Cre retina. Rows **A, B**, control sections (row **A**) and *Dync1h1*^F/F^;Six3Cre knockout sections (row **B**) probed with anti-calbindin recognizing horizontal, amacrine and ganglion cells (GCs). Note at P12, several neurons have survived due to persistent expression of DYNC1H1 in the IPL. Rows **C, D**, control sections (row C) and *Dync1h1*^F/F^;Six3Cre knockout sections (row D) probed with anti-PKCα, a marker for rod bipolar cells (BPs). Note location of rod BP nuclei at the sclerad INL border in row C, and the scattered distribution of nuclei in row D.

Persistence of DYNC1H1 in the mutant IPL (Fig 3, row **B**) prompted us to identify surviving cells of the inner retina (horizontal, rod bipolar, amacrine, ganglion cells and Müller glia). Calbindin, a soluble Ca^2+^-binding protein, serves as a marker for horizontal (HCs), amacrine and ganglion cells (GCs) [31]. HCs are located at the sclerad INL of control retina (Fig 5, row **A**). In the P8 mutant retina, calbindin-positive horizontal and amacrine neurons appeared fewer in number. At P12 when photoreceptors have degenerated, several calbindin-positive neurons survived (Fig 5, row **B**). We tested for presence of rod bipolar cells (BPs) employing anti-PKCα, a protein kinase abundantly expressed in rod BP dendrites, cell bodies and axons (Fig 5, row **C**) [32]. Rod synapses contact BP dendrites to transmit the scotopic light impulse to ganglion cells. At P6 and later, BP nuclei are located at the sclerad edge of the INL; by contrast, *Dync1h1*^F/F^;Six3Cre BP nuclei are scattered throughout the INL (Fig 5, row **D**).

Müller glia cells (MGCs) are the last-born and last postmitotic cell type of the mammalian retina [33]. To identify the fate of MGCs in the *Dync1h1*^F/F^;Six3Cre retina, we employed a glutamine synthetase antibody in P6-P12 cryosections (S1 Fig). The results show that while MGCs in control sections are undetectable at P6, MGC endfoot structures form around P8. MGCs extend processes into the ONL by P10 and microvilli surrounding the IS can be detected by P14 (S1 Fig, row **A**). Nuclei are precisely centered in the INL by P10. In *Dync1h1*^F/F^;Six3Cre retina, endfoot structures, microvilli and processes are absent (S1 Fig, row **B**). We conclude that functional MGCs never form the *Dync1h1*^F/F^;Six3Cre retina, and that persistent DYNC1H1 expression in the mutant IPL mostly originates from remaining amacrine, BP and ganglion cells.

### Cone survival in *Dync1h1*^F/F^;Six3Cre central retina

**Fig 6:**
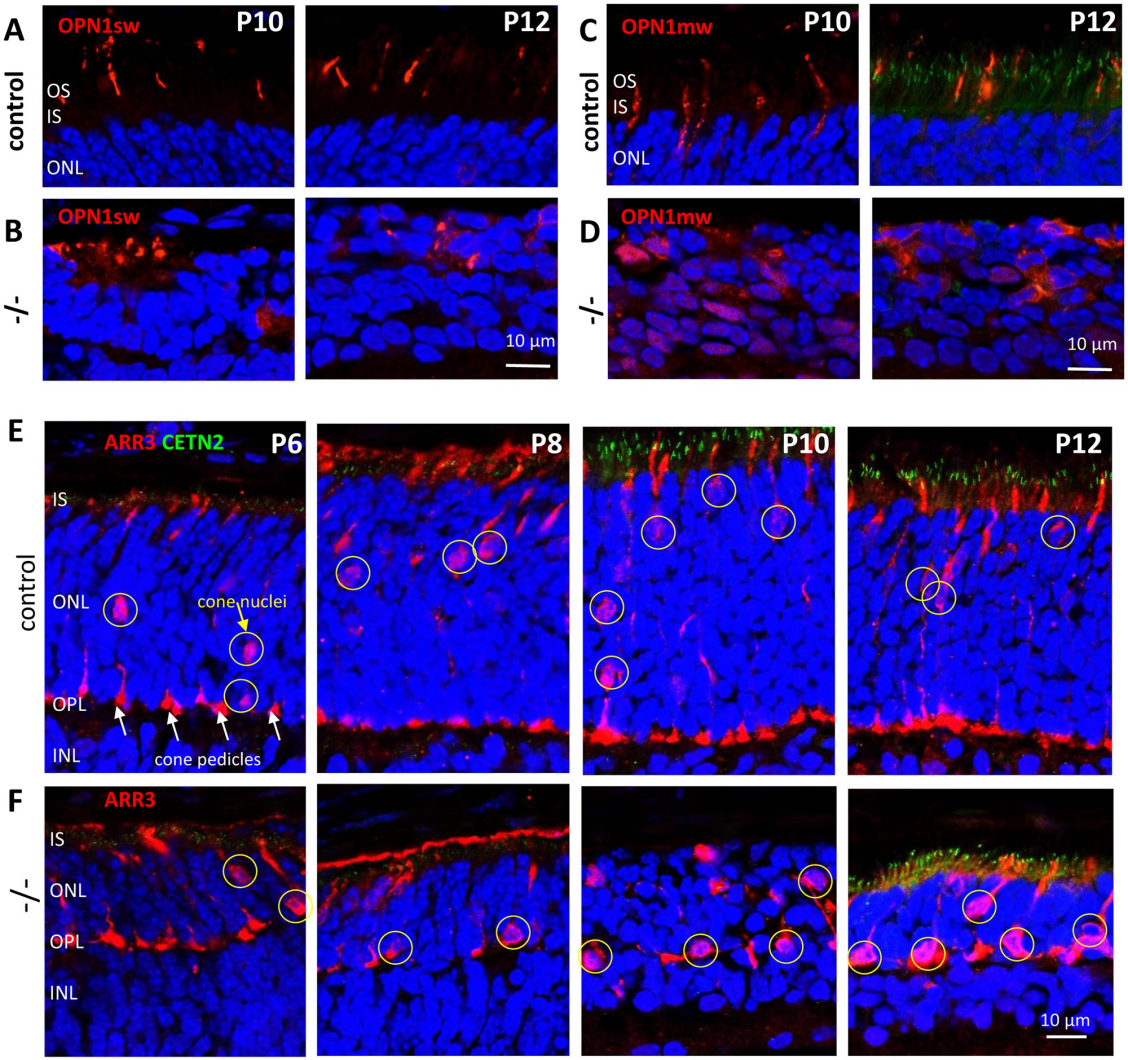
*Dync1h1*^F/F^;Six3Cre cones produce pedicles and axons but no outer segments. Rows **A, B,** control sections (row A) and knockout sections (row B) probed with anti-S-opsin at P10-P12. Rows **C, D,** control sections (row C) and knockout sections (row D) probed with anti-ML-opsin at P10-12. S-opsin and M-opsin are sequestered in the mutant ONL and cone OS are not elaborated. Rows **E, F**, control sections (row **E**) and knockout sections (row **F**) probed with anti-cone arrestin at P6-P12. Cone arrestin diffuses freely and labels pedicles (indicated by white arrows), axons, and inner segments. Cone nuclei (indicated by yellow circles) are scattered in the ONL, and slowly move towards the apical ONL by P12 in control. Mutant cone cells are remarkably stable through P12, but nuclei are adjacent to the OPL. OS (row **E**) are not visible, as the retina was not exposed to light.

To investigate the fate of cones in the absence of dynein, we employed anti-S-opsin, anti-ML-opsin, and anti-cone arrestin as cone markers. Mouse cones express two visual pigments in most cones, M-opsin and S-opsin, and both localize exclusively to the cone OS. Cone arrestin (ARR3) is a soluble protein that freely diffuses through the cell, thereby visualizing synaptic pedicles, cone nuclei and cone dendrite/axons. Cone OS arise as early as P4 and cone pedicles around P7 [34]. In control sections, cone OS are weakly detectable at P6 (not shown) and rise to near full length by P12 (Fig 6, rows **A, C**). In knockout sections, S-opsin and M-opsin aggregate near the edge of the nuclear layer and OS are not formed (Fig 6, rows **B, D**).

Cone nuclei are scattered throughout the ONL at P6 but move toward the OLM by P12, presumably pulled by dynein [35, 36]. In our control sections, cone nuclei had mostly reached their final position by P12 (Fig 6, row **E**). In contrast, mutant nuclei are positioned mostly near the OPL edge (Fig 6, row **F**, P12). Synaptic enlargements representing developing cone pedicles can be observed as early as P6 in control sections (Fig 6, row **E**), clearly marking the OPL. In *Dync1h1*^F/F^;Six3Cre retina, developing mutant cone pedicles are formed allowing delineation of the mutant OPL (Fig 6, row **F**). Mutant pedicles, however, do not contain CtBP2/Ribeye, suggesting dynein may be necessary for its correct positioning.

#### Preserved retina lamination and function at the peripheral retina

**Fig 7:**
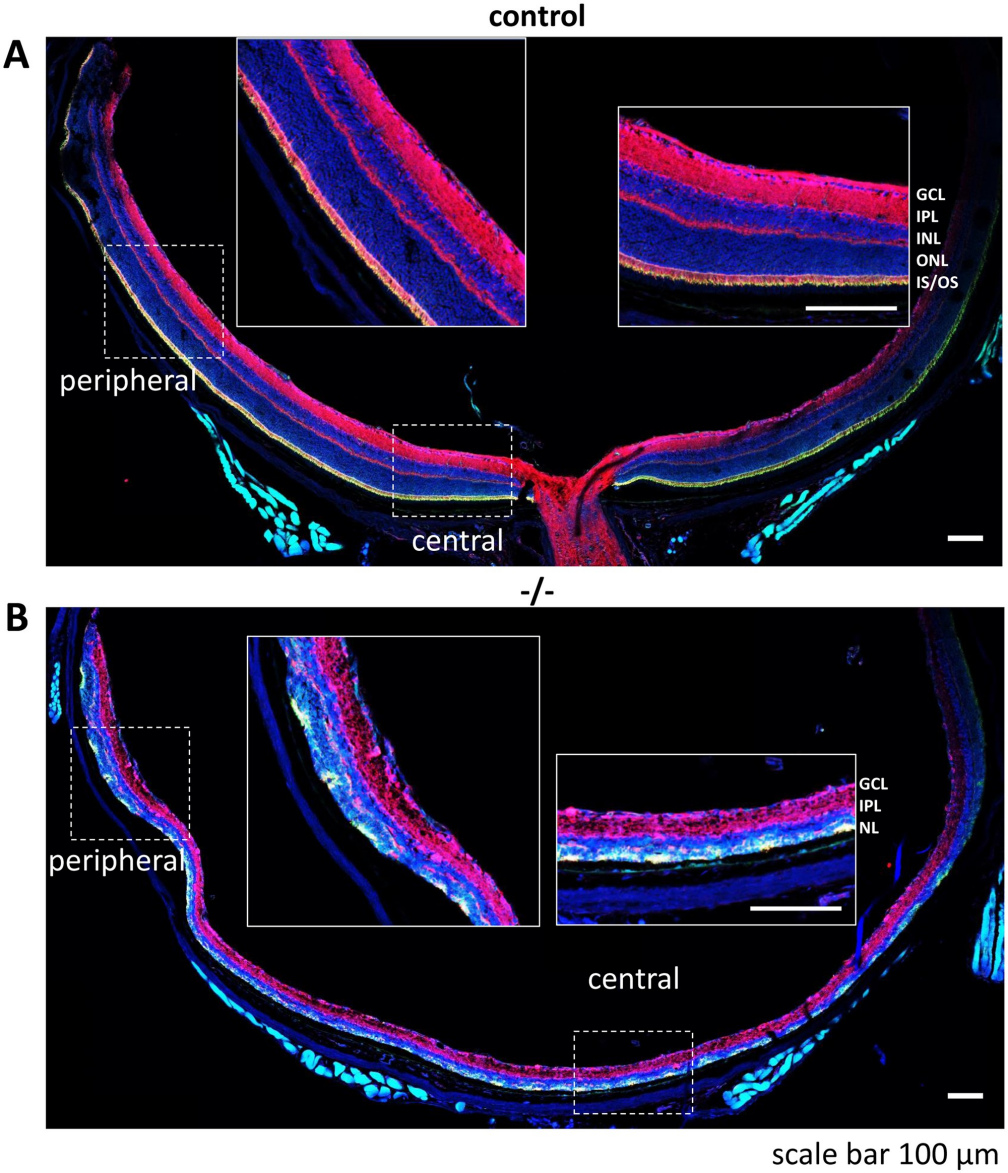
Control and *Dync1h1*^F/F^;Six3Cre retina cross sections. **A, B,** retina cross section of *Dync1h1*^*F/F*^ (A) and *Dync1h1*^F/F^;Six3Cre mice (B) labeled with DAPI (blue), anti-DYNC1H1 (red), and EGFP-CETN2 (green). Peripheral and central enlargements are screen shots taken at 54% zoom (ZEN software). Note persistent but reduced presence of DYNC1H1 in the peripheral *Dync1h1*^F/F^;Six3Cre retina (B), and degeneration of central retina photoreceptors. Scale bars, 100 μm.

Mitosis ceases in the central retina by P6, and in the peripheral retina by P11 [37]. Further, Six3Cre drives Cre recombinase expression more thoroughly in the central than in the peripheral retina [24]. Consequently, lamination of the nuclear layer and morphogenesis of inner and outer segments can be observed at the peripheral edge of the *Dync1h1*^F/F^;Six3Cre retina while the central retina is degenerated. This is demonstrated by comparing a *Dync1h1* ^F/F^ control cross section retina with a knockout at P16 (Fig 7). The control retina shows consistent expression of DYNC1H1 in the IPL, OPL, and IS of the center and periphery (Fig 7A). By contrast, the *Dync1h1*^F/F^;Six3Cre central retina shows low and patchy presence of DYNC1H1 within the merged ONL/INL or at the sclerad edge of the ONL; the peripheral retina is less affected. Presence of DYNC1H1 in the P16 IPL persists in both peripheral and central retina (Fig 7B).

**Fig 8:**
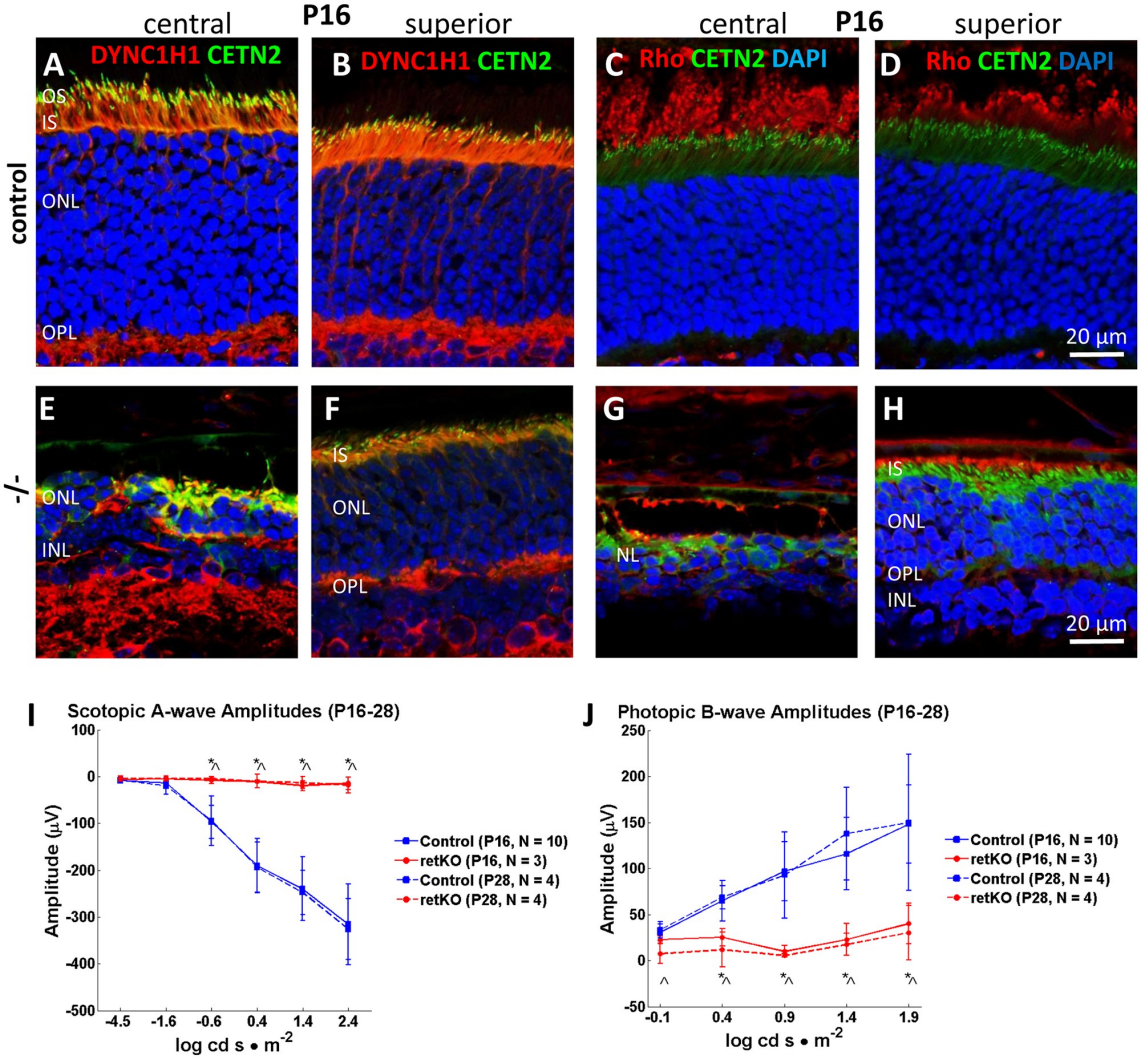
Central versus superior peripheral phenotypes at P16. **A-H,** P16 central (A, C, E, G) and superior (B, D, F, H) cryosections of control (A-D) and knockout retina (E-H) probed with anti-DYNC1H1 (A, B, E, F) and rhodopsin antibody (C, D, G, H). Note central knockout retina (E, G) is severely deteriorated while superior retina (F, H) is relatively well preserved with 7–8 rows of nuclei. **I, J,** ERGs measuring pan-retina scotopic a-wave (I) and photopic b-wave (J) amplitudes at P16 and P28. The scotopic response is nearly extinguished whereas high-intensity flashes elicit a low photopic b-wave response. Blue lines, control (solid line, P16; hatched line, P28). Red lines, *Dync1h1*^F/F^;Six3Cre (solid lines, P16; hatched lines, P28). n = number of animals. Two-factor ANOVA with post-hoc multiple comparison by Tukey’s honestly significant difference criterion, ^ indicates p<0.05, P16; * indicates p<0.05, P28.

Extended survival of P16 peripheral retina is further demonstrated by IHC analysis of control and *Dync1h1*^F/F^;Six3Cre retina sections. ONL thickness of control central and superior retina is similar at P16 (Fig 8A–8D). The central *Dync1h1*^F/F^;Six3Cre retina is nearly completely degenerated at this age (Fig 8E and 8G), but the *Dync1h1*^F/F^;Six3Cre superior retina still forms ONL, INL and OPL layers (Fig 8F and 8H). DYNC1H1 is much reduced in the superior *Dync1h1*^F/F^;Six3Cre IS (Fig 8F), and short OS are present as evidenced by presence of rhodopsin (Fig 8H). Formation of OS in the retina periphery encouraged analysis of function by pan-retina ERG. Scotopic a-wave amplitudes are almost extinguished at P16 and later (Fig 8I). Flashes at 1.4 log cd s·m^-2^ and higher under photopic conditions elicit a b-wave response (20% of normal) (Fig 8J) suggesting cone survival at the periphery.

## Discussion

In this communication, we truncated the dynein heavy chain after exon 23 removing the motor and the microtubule-binding domain. Truncation of the heavy chain is expected to eliminate the function of dynein, with drastic consequences on membrane protein trafficking. The fate of the N-terminal fragment encompassing exons 1–23 has no motor function and was not investigated for lack of N-terminal antibodies. However, since heterozygous animals are phenotypically normal on both ERG and immunohistochemistry, we assume the presence or absence of truncated protein does not affect the knockout phenotype. In control animals, DYNC1H1 is prominently expressed in photoreceptor IS, OPL and IPL, and to a lesser extent within the ONL and INL (Fig 3, row **A**). In conditional knockouts, DYNC1H1 could not be detected in the IS area but trace amounts persisted at the distal edge or within the nuclear layer (Fig 3, row **B**). These pockets also contained traces of rhodopsin, cone pigments, PDE6, EGFP-CETN2 labeled centrosomes and presumably other photoreceptor proteins (Fig 4). Photoreceptors do not elaborate IS, OS or functional synaptic terminals and degenerate in the first two postnatal weeks. Cones apparently form axons connecting to pedicles but are nonfunctional (Fig 6).

We studied the fate of two rod phototransduction proteins, rhodopsin and PDE6, in the *Dync1h1*^F/F^;Six3Cre retina. Rhodopsin is a G-protein-coupled receptor [38, 39] that is synthesized at the rough ER surrounding the rod nuclei and known to depend on dynein for transport to the Golgi [5, 40] following the conventional secretory pathway [3, 41]. Transport of vesicles terminates near the basal body at the periciliary membrane [4] where cargo is assembled for transport through the connecting cilium. In P6 control retina, rhodopsin is located in the distal IS and is found in budding OS by P8 (Fig 4, row **A**). In the *Dync1h1*^F/F^;Six3Cre retina, rhodopsin aggregated near centrosomes marked by EGFP-CETN2 at the sclerad edge of the ONL (Fig 4, rows **B** and **B’**). Unexpectedly, rhodopsin did not accumulate at the rod perinuclear rough ER at P6 suggesting suppression of opsin synthesis at a time when OS should form.

Rod and cone PDE6 are peripheral membrane proteins attached to membranes by C-terminal isoprenylation [42–44] and traffic to the OS by diffusion independent of dynein. Diffusion likely occurs via a complex with the prenyl binding protein PDE6D (PDEδ) [27, 45]. In the mutant retina at P6, PDE6 is deposited at the ONL sclerad edge, presumably membrane bound (Fig 4, row **D**). At P8-P12, PDE6-positive cells are collapsing into foci rich in CETN2, centrosomes and presumably other soluble proteins. Interestingly, a few mother centrioles (basal bodies) appear to be able to engage in ciliogenesis and form diminutive membrane OS-like structures filled with PDE6. In zebrafish *cnb* and *mok* mutants, basal body docking was unaffected supporting this result [46]. As MOE antibody recognizes both rod and cone PDE6 it is not possible to distinguish rod and cone OS, but based on structural integrity of cones (Fig 6) we interpret these structures to originate from cones. *Dync1h1*^F/F^;Six3Cre central rods are degenerated at P6 (Fig 4), *Dync1h1*^F/F^;Six3Cre cones still formed pedicles connected to nuclei by axons through P12 (Fig 6, row **B**).

Another major observation in the knockout model is the nuclear layer disorganization beginning at P6, before OS are formed, most likely caused by nuclear mispositioning. Active nuclear positioning in the retina takes place in the first two postnatal weeks and is managed by two motors, dynein and kinesin-2, walking towards the minus end and plus end of microtubules respectively [47]. In P4 WT retina, the neuroblastic layer is uniform and does not show an OPL with synaptic connections [48]. At P6, the *Dync1h1*^F/F^;Six3Cre OPL could not easily be distinguished (Figs 2–5). The nuclear layer was often interrupted suggesting that nuclei were not positioned correctly, a phenotype consistent with polarity defects [49–51]. Similar disorganization is observed when Syne2/Nesprin2 and Sun2, members of the LINC complex mediating nuclear migration during retina development, are deleted [36, 52].

All retinal cell types arise from a pool of multipotent progenitors [53]. Expression of Cre beginning at E9 affects the entire developing retina and RPE by E12.5 [23]. In mouse, central retina neurogenesis completes by P5 and ciliogenesis begins at P6. We observed that control and *Dync1h1*^F/F^;Six3Cre basal bodies indeed formed a CC at P6, a rare event at this early stage, but at P8 ciliogenesis discontinued in *Dync1h1*^F/F^;Six3Cre retina (Fig 2E–2H). The 25% reduction in the neuronal layer at P6 (Fig 3E) suggests reduced neurogenesis and/or increased cell death during development in the absence of dynein. This reduction is consistent with the role of dynein in mitotic cell division such as spindle orientation and assembly, nuclear migration, chromosome segregation and cytokinesis (see review by [54, 55]) as well as in neuronal survival [56]. Surprisingly, DYNC1H1 deletion did not affect cells in the retina uniformly. While the *Dync1h1* gene is completely knocked out at P6 (Fig 1G), the mutant IPL continued to harbor DYNC1H1 polypeptides (Fig 3B and 3B’) through P16 (Fig 7B) suggesting that inner retina may be much less effective than photoreceptors in eliminating “old” heavy chains synthesized before *Dync1h1* gene knockout. We believe this accounts for the survival of inner retina neurons through P16 (Fig 5, row **B**, **D**).

## Material and methods

### Animals

All procedures were approved by the University of Utah Institutional Animal Care and Use Committee (Protocol 18–11005). Mice were sacrificed by cervical dislocation. For ERG studies, mice were anesthetized with intraperitoneal injection of 1% ketamine/0.1% xylazine at 10 μl/g body weight.

### Generation of the *Dync1h1* conditional allele

A cell line (*Dync1h1*^tm1a(KOMP)^) in which a gene trap was placed in intron 23, and a loxP site in intron 25 (Fig 1), was obtained from the UC-Davis KOMP repository. Chimeric mice were produced by the University of Utah Transgenic Gene Targeting Mouse Facility by blastocyst injection. Germline transmission of the *Dync1h1*^GT^ allele was verified by PCR using primers upstream and downstream of the loxP site in intron 25 (see genotyping paragraph). Founder mice were bred with flp-recombinase transgenic mice to create the *Dync1h1*^F^ allele. Mice carrying the *Dync1h1*^F^ allele were outbred to C57BL/6J mice to remove the rd8 mutation [57]. *Dync1h1*^F/F^ were crossed with Six3Cre transgenic mice (Stock No: 019755, The Jackson Laboratory) which were Egfp-*Cetn2* positive (JAX 008234—CB6-Tg(CAG-EGFP/CETN2)3-4Jgg/J) [58] to generate *Dync1h1* ^F/+^;Six3Cre;Egfp-*Cetn2* mice. Mice were then crossed back to *Dync1h1*^F/F^ to generate experimental animals. Expression of Egfp-Cetn2 allows centriole identification without use of a specific antibody. All transgenic mice (Six3Cre, flp, Egfp-*Cetn2*) were outbred routinely to C57BL/6J mice.

### Genotyping

DNA was extracted from fresh tissue (tail or retina) by dissolving tail clips or entire retina from P4-14 day old mice in 100–200 μL tail lysis buffer at 50–60°C for 1–2 hour. Digests were then centrifuged at 15000 rpm for 5 minutes. Supernatant was added to an equal volume of isopropanol and centrifuged at 15000 rpm for 5 minutes. Pellet was rehydrated in 100 μL H_2_O. Genotyping was done by polymerase chain reaction with EconoTaq^®^ DNA polymerase (Lucigen) using the following primers. Deletion of exons 24 and 25 was verified by amplification with primers exon 23-F (5’-TCTCTGGAAAGGTTGGCAGA) and loxP-R (sequence below) using P6 retina genomic DNA as template. Amplicon sizes are WT allele (1.3 kb) and Dync1h1^F^ allele (1.6 kb) for the Dync1h1^F/+^ control, and cre-recombination allele (500 bp) for Dync1h1^F/F^;Six3Cre (P6) (Fig 1F). Presence of loxP in intron 25 was verified by PCR using primers loxP-F (5’-TGATGGTCTTGGCTAATTGGTGG) and loxP-R (5’-GAGATCAGTTGCGGTTTGCTAGT) with tail DNA as template, yielding amplicon sizes of 269 bp (floxed) and 202 bp (WT) (Fig 1G). Presence of Six3Cre transgene was verified with Six3Cre-F (5’-TCGATGCAACGAGTGATGAG); Six3Cre-R (5’-TTCGGCTATACGTAACAGGG) (amplicon size 450 bp) (Fig 1H). Presence of EGFP-CETN2 was determined by PCR using primers Cetn2-F (5’-TGAACGAAATCTTCCCAGTTTCA) and Cetn2-R (5’-ACTTCAAGATCCGCCACAACAT) (amplicon size 600 bp) (Fig 1I).

### Electroretinography

Scotopic and photopic ERG was performed using P16 and P28 mice. Prior to ERG the mice were dark-adapted overnight and anesthetized with intraperitoneal injection of 1% ketamine/0.1% xylazine at 100 μl/g body weight. The mice were kept warm during ERG by using a temperature-controlled stage. Scotopic and photopic responses were recorded as described [59] using a UTAS BigShot Ganzfeld system (LKC Technologies, Gaithersburg, MD). Scotopic single-flash responses were recorded at stimulus intensities of -4.5 log cd s·m^-2^ (log candela seconds per square meter) to 2.4 log cd s·m^-2^). Mice were light-adapted under a background light of 1.48 log cd s·m^-2^ for 5–10 min prior to measuring photopic responses. Single-flash responses of control and knockout were recorded at stimulus intensities of -0.1 log cd s·m^-2^ to 1.9 log cd s·m^-2^.

### Eye collection methods

Mice were sacrificed by cervical dislocation. Eyes were enucleated and fixed by immersion in 4% paraformaldehyde in 0.1M phosphate buffer, pH 7.4, for 1 hour. Anterior segments were removed after 10 minutes of fixation. Eyecups were equilibrated in 15% sucrose in PBS, then equilibrated overnight in 30% sucrose in PBS, embedded in OCT, frozen on dry ice and stored at -80°C. Blocks containing eyecups were equilibrated to -20°C prior to sectioning. Transverse sections, including or adjacent to the optic nerve, were cut at 14μm using a Leica cryostat and transferred to charged slides (Thermo-Fisher). Slides were stored at -80°C. Control retinas came from *Dync1h1* ^F/F^, *Dync1h1* ^F/+^, or *Dync1h1* ^F/+^;Six3Cre mice.

### Immunohistochemistry

Sections were encircled using a PAP pen (Ted Pella), warmed for 30 minutes at 37°C, then rehydrated by washing 10 min X3 in 1X PBS. Unless otherwise noted, all sections used were from the central retina near the optic nerve head. Sections were blocked in 5% normal goat serum (NGS)/0.1% TritonX-100 in 1X PBS for 1 hour. Antibodies, dilutions and sources follow: DYNC1H1 (dilution 1:250, Proteintech 12345-1-AP) [7, 60]; rhodopsin (1:1000, 4D2 Abcam 98887); PDE6 (1:1000, MOE Cytosignal) [45]; OPN1SW (1:500, Millipore Sigma AB5407) [59, 61]; OPN1MW/OPN1LW (1:500, Millipore Sigma AB5405) [45, 62]; cone arrestin (ARR3, 1:250, outsourced to Covance, Princeton NJ and purified in house) [63]; CtBP2/Ribeye (1:10,000, BD Biosciences 612044); Calbindin (1:200, Proteintech 14479-1-AP); PKCα (1:150, Cell Signaling 20565); monoclonal anti-Glutamine Synthetase (GluSyn), BD Biosciences 610518). Primary antibodies were diluted in blocking buffer and applied to sections; sections were then incubated overnight at 4°C. Slides were washed for 10 min X3 in PBS. Secondary antibodies were diluted in blocking buffer (goat anti-mouse Alexa Fluor 555, 1:1200 (Invitrogen 32737); goat anti-rabbit Alexa Fluor 555, 1:1000; (Invitrogen 32732), DAPI, 1:5000), applied to the sections and sections were incubated in the dark for 1 hour at room temperature. Slides were washed for 10 min X3 in PBS. Slides were dipped briefly in deionized H_2_O, and coverslipped using Fluoromount-G^®^ Mounting Medium (Southern Biotech). Images were acquired using a Zeiss LSM800 confocal microscope and either the 40X (most sections), 60X (Opn1sw & Opn1mw), or 20X (retina cross sections) objective. All genotypes of a given age and antibody were imaged at a single z-plane using identical settings for laser intensity and master gain. Digital gain was 1 for all images. Pinhole size was set for 1AU on the red channel (39 μm for the 40X objective). Tiled retina cross section images were compiled with 10% overlap between individual frames. All micrographs shown in Figs 3–8 are representative of at least triplicate experiments. Post-processing of non-saturated images consisted of equal adjustments to brightness and contrast of control and knockout images using Adobe Photoshop but did not affect the conclusions drawn.

### Statistical analysis

We performed an unbalanced two-factor ANOVA to compare experimental and control animals for their quantified A- and B-wave ERG response across multiple ages. Post-hoc multiple comparison was performed using Tukey’s honestly significant difference criterion. Statistical significance was determined using an alpha value of p < 0.05. All electroretinography statistics were computed using MATLAB’s statistical toolbox "anovan" and "multcompare" functions.

We performed a Student’s *t*-Test assuming unequal variances to compare control and KO central retinal thicknesses, measured just superior and inferior of the optic nerve on fixed retinal slices. Microsoft Excel function "T.TEST assuming unequal variances" was used to calculate the p-value, with statistical significance determined using an alpha value of p < 0.05.

## Supporting information

S1 Fig Müller glia cells (MGC). Rows **A, B**, control sections (row A) and *Dync1h1*^F/F^;Six3Cre knockout sections (row B) probed with anti-glutamine synthetase (GluSyn) recognizing Müller Glia with nuclei centered in the INL. Control MGC endfoot structures start to develop at P8 and processes reach their full extension to the OLM by P14. At P16 few GluSyn-positive nuclei are seen in *Dync1h1*^F/F^;Six3Cre (arrow in row **B**).
(TIF)

S1 Raw images (PDF)

## References

[1] Young RW. The renewal of photoreceptor cell outer segments. The Journal of Cell Biology. 1967;33:61–72. 10.1083/jcb.33.1.61 6033942 PMC2107286

[2] Young RW. Visual cells and the concept of renewal. Investigative Ophthalmology & Visual Science. 1976;15(9):700–25. 986765

[3] van Vliet C, Thomas EC, Merino-Trigo A, Teasdale RD, Gleeson PA. Intracellular sorting and transport of proteins. Prog Biophys Mol Biol. 2003;83(1):1–45. 10.1016/s0079-6107(03)00019-1 12757749

[4] Nemet I, Ropelewski P, Imanishi Y. Rhodopsin Trafficking and Mistrafficking: Signals, Molecular Components, and Mechanisms. Prog Mol Biol Transl Sci. 2015;132:39–71. 10.1016/bs.pmbts.2015.02.007 26055054

[5] Tai AW, Chuang JZ, Bode C, Wolfrum U, Sung CH. Rhodopsin’s carboxy-terminal cytoplasmic tail acts as a membrane receptor for cytoplasmic dynein by binding to the dynein light chain Tctex-1. Cell. 1999;97(7):877–87. 10.1016/s0092-8674(00)80800-4 10399916

[6] Lewis TR, Zareba M, Link BA, Besharse JC. Cone myoid elongation involves unidirectional microtubule movement mediated by dynein-1. Mol Biol Cell. 2018;29(2):180–90. 10.1091/mbc.E17-08-0525 29142075 PMC5909930

[7] Insinna C, Baye LM, Amsterdam A, Besharse JC, Link BA. Analysis of a zebrafish dync1h1 mutant reveals multiple functions for cytoplasmic dynein 1 during retinal photoreceptor development. Neural Dev. 2010;5:12. 10.1186/1749-8104-5-12 20412557 PMC2880287

[8] Grotjahn DA, Lander GC. Setting the dynein motor in motion: New insights from electron tomography. J Biol Chem. 2019;294(36):13202–17. 10.1074/jbc.REV119.003095 31285262 PMC6737236

[9] Carter AP, Diamant AG, Urnavicius L. How dynein and dynactin transport cargos: a structural perspective. Curr Opin Struct Biol. 2016;37:62–70. 10.1016/j.sbi.2015.12.003 26773477

[10] Allan VJ. Cytoplasmic dynein. Biochemical Society transactions. 2011;39(5):1169–78. 10.1042/BST0391169 21936784

[11] Tsujikawa M, Omori Y, Biyanwila J, Malicki J. Mechanism of positioning the cell nucleus in vertebrate photoreceptors. Proc Natl Acad Sci U S A. 2007;104(37):14819–24. 10.1073/pnas.0700178104 17785424 PMC1976238

[12] Harada A, Takei Y, Kanai Y, Tanaka Y, Nonaka S, Hirokawa N. Golgi vesiculation and lysosome dispersion in cells lacking cytoplasmic dynein. J Cell Biol. 1998;141(1):51–9. 10.1083/jcb.141.1.51 9531547 PMC2132725

[13] Courchesne SL, Pazyra-Murphy MF, Lee DJ, Segal RA. Neuromuscular junction defects in mice with mutation of dynein heavy chain 1. PLoS One. 2011;6(2):e16753. 10.1371/journal.pone.0016753 21346813 PMC3035627

[14] Hafezparast M, Klocke R, Ruhrberg C, Marquardt A, Ahmad-Annuar A, Bowen S, et al. Mutations in dynein link motor neuron degeneration to defects in retrograde transport. Science. 2003;300(5620):808–12. 10.1126/science.1083129 12730604

[15] Ori-McKenney KM, Vallee RB. Neuronal migration defects in the Loa dynein mutant mouse. Neural Dev. 2011;6:26. 10.1186/1749-8104-6-26 21612657 PMC3127822

[16] Hafezparast M, Ahmad-Annuar A, Hummerich H, Shah P, Ford M, Baker C, et al. Paradigms for the identification of new genes in motor neuron degeneration. Amyotroph Lateral Scler Other Motor Neuron Disord. 2003;4(4):249–57. 10.1080/14660820310016084 14753659

[17] Harms MB, Ori-McKenney KM, Scoto M, Tuck EP, Bell S, Ma D, et al. Mutations in the tail domain of DYNC1H1 cause dominant spinal muscular atrophy. Neurology. 2012;78(22):1714–20. 10.1212/WNL.0b013e3182556c05 22459677 PMC3359582

[18] Scoto M, Rossor AM, Harms MB, Cirak S, Calissano M, Robb S, et al. Novel mutations expand the clinical spectrum of DYNC1H1-associated spinal muscular atrophy. Neurology. 2015;84(7):668–79. 10.1212/WNL.0000000000001269 25609763 PMC4336105

[19] Nandini S, Conley Calderon JL, Sabblah TT, Love R, King LE, King SJ. Mice with an autosomal dominant Charcot-Marie-Tooth type 2O disease mutation in both dynein alleles display severe moto-sensory phenotypes. Sci Rep. 2019;9(1):11979. 10.1038/s41598-019-48431-7 31427617 PMC6700207

[20] Sabblah TT, Nandini S, Ledray AP, Pasos J, Calderon JLC, Love R, et al. A novel mouse model carrying a human cytoplasmic dynein mutation shows motor behavior deficits consistent with Charcot-Marie-Tooth type 2O disease. Sci Rep. 2018;8(1):1739. 10.1038/s41598-018-20081-1 29379136 PMC5789002

[21] Schiavo G, Greensmith L, Hafezparast M, Fisher EM. Cytoplasmic dynein heavy chain: the servant of many masters. Trends Neurosci. 2013;36(11):641–51. 10.1016/j.tins.2013.08.001 24035135 PMC3824068

[22] Skarnes WC, Rosen B, West AP, Koutsourakis M, Bushell W, Iyer V, et al. A conditional knockout resource for the genome-wide study of mouse gene function. Nature. 2011;474(7351):337–42. 10.1038/nature10163 21677750 PMC3572410

[23] Furuta Y, Lagutin O, Hogan BL, Oliver GC. Retina- and ventral forebrain-specific Cre recombinase activity in transgenic mice. Genesis. 2000;26(2):130–2. 10686607

[24] Diacou R, Zhao Y, Zheng D, Cvekl A, Liu W. Six3 and Six6 Are Jointly Required for the Maintenance of Multipotent Retinal Progenitors through Both Positive and Negative Regulation. Cell Rep. 2018;25(9):2510–23 e4. 10.1016/j.celrep.2018.10.106 30485816 PMC6317371

[25] Vilchez D, Saez I, Dillin A. The role of protein clearance mechanisms in organismal ageing and age-related diseases. Nat Commun. 2014;5:5659. 10.1038/ncomms6659 25482515

[26] Wassle H, Puller C, Muller F, Haverkamp S. Cone contacts, mosaics, and territories of bipolar cells in the mouse retina. J Neurosci. 2009;29(1):106–17. 10.1523/JNEUROSCI.4442-08.2009 19129389 PMC6664901

[27] Zhang H, Li S, Doan T, Rieke F, Detwiler PB, Frederick JM, et al. Deletion of PrBP/delta impedes transport of GRK1 and PDE6 catalytic subunits to photoreceptor outer segments. Proc Natl Acad Sci U S A. 2007;104(21):8857–62. 10.1073/pnas.0701681104 17496142 PMC1885592

[28] Avasthi P, Watt CB, Williams DS, Le YZ, Li S, Chen CK, et al. Trafficking of membrane proteins to cone but not rod outer segments is dependent on heterotrimeric kinesin-II. J Neurosci. 2009;29(45):14287–98. 10.1523/JNEUROSCI.3976-09.2009 19906976 PMC2788486

[29] Frederick JM, Krasnoperova NV, Hoffmann K, Church-Kopish J, Ruther K, Howes K, et al. Mutant rhodopsin transgene expression on a null background. Invest Ophthalmol Vis Sci. 2001;42(3):826–33. 11222546

[30] Lem J, Krasnoperova NV, Calvert PD, Kosaras B, Cameron DA, Nicol, et al. Morphological, physiological, and biochemical changes in rhodopsin knockout mice. Proc Natl Acad Sci U S A. 1999;96(2):736–41. 10.1073/pnas.96.2.736 9892703 PMC15206

[31] Mitchell CK, Rowe-Rendleman CL, Ashraf S, Redburn DA. Calbindin immunoreactivity of horizontal cells in the developing rabbit retina. Exp Eye Res. 1995;61(6):691–8. 10.1016/s0014-4835(05)80020-x 8846841

[32] Haverkamp S, Ghosh KK, Hirano AA, Wassle H. Immunocytochemical description of five bipolar cell types of the mouse retina. J Comp Neurol. 2003;455(4):463–76. 10.1002/cne.10491 12508320 PMC2834891

[33] Vecino E, Rodriguez FD, Ruzafa N, Pereiro X, Sharma SC. Glia-neuron interactions in the mammalian retina. Prog Retin Eye Res. 2016;51:1–40. 10.1016/j.preteyeres.2015.06.003 26113209

[34] Fei Y. Development of the cone photoreceptor mosaic in the mouse retina revealed by fluorescent cones in transgenic mice. Mol Vis. 2003;9:31–42. 12592228

[35] Rich KA, Zhan YT, Blanks JC. Migration and synaptogenesis of cone photoreceptors in the developing mouse retina. J Comp Neurol. 1997;388:47–63. 9364238

[36] Razafsky D, Blecher N, Markov A, Stewart-Hutchinson PJ, Hodzic D. LINC complexes mediate the positioning of cone photoreceptor nuclei in mouse retina. PLoS One. 2012;7(10):e47180. 10.1371/journal.pone.0047180 23071752 PMC3465324

[37] Young RW. Cell differentiation in the retina of the mouse. The Anatomical Record. 1985;212:199–205. 10.1002/ar.1092120215 3842042

[38] Palczewski K, Kumasaka T, Hori T, Behnke CA, Motoshima H, Fox BA, et al. Crystal structure of rhodopsin: A G protein-coupled receptor [see comments]. Science. 2000;289(5480):739–45. 10.1126/science.289.5480.739 10926528

[39] HArgrave PA. Rhodopsin chemistry, structure, and topography. Progr Ret Res. 1982;1:1–51.

[40] Kong S, Du X, Peng C, Wu Y, Li H, Jin X, et al. Dlic1 deficiency impairs ciliogenesis of photoreceptors by destabilizing dynein. Cell Res. 2013;23(6):835–50. 10.1038/cr.2013.59 23628724 PMC3674393

[41] Barlowe CK, Miller EA. Secretory protein biogenesis and traffic in the early secretory pathway. Genetics. 2013;193(2):383–410. 10.1534/genetics.112.142810 23396477 PMC3567731

[42] Qin N, Pittler SJ, Baehr W. In vitro isoprenylation and membrane association of mouse rod photoreceptor cGMP phosphodiesterase alpha and beta subunits expressed in bacteria. J Biol Chem. 1992;267(12):8458–63. 1314827

[43] Anant JS, Ong OC, Xie H, Clarke S, O’Brien PJ, Fung BK-K. In vivo differential prenylation of retinal cyclic GMP phosphodiesterase catalytic subunits. J Biol Chem. 1992;267:687–90. 1309771

[44] Li TS, Volpp K, Applebury ML. Bovine cone photoreceptor cGMP phosphodiesterase structure deduced from a cDNA clone. Proc Natl Acad Sci U S A. 1990;87(1):293–7. 10.1073/pnas.87.1.293 2153291 PMC53249

[45] Hanke-Gogokhia C, Wu Z, Gerstner CD, Frederick JM, Zhang H, Baehr W. Arf-like Protein 3 (ARL3) Regulates Protein Trafficking and Ciliogenesis in Mouse Photoreceptors. J Biol Chem. 2016;291(13):7142–55. 10.1074/jbc.M115.710954 26814127 PMC4807295

[46] Fogerty J, Denton K, Perkins BD. Mutations in the Dynein1 Complex are Permissible for Basal Body Migration in Photoreceptors but Alter Rab6 Localization. Adv Exp Med Biol. 2016;854:209–15. 10.1007/978-3-319-17121-0_28 26427413 PMC4939795

[47] Baye LM, Link BA. Nuclear migration during retinal development. Brain Res. 2008;1192:29–36. 10.1016/j.brainres.2007.05.021 17560964 PMC2674389

[48] Song H, Bush RA, Vijayasarathy C, Fariss RN, Kjellstrom S, Sieving PA. Transgenic expression of constitutively active RAC1 disrupts mouse rod morphogenesis. Invest Ophthalmol Vis Sci. 2014;55(4):2659–68. 10.1167/iovs.13-13649 24651551 PMC4001786

[49] Wei X, Malicki J. nagie oko, encoding a MAGUK-family protein, is essential for cellular patterning of the retina. Nat Genet. 2002;31(2):150–7. 10.1038/ng883 11992120

[50] Masai I, Lele Z, Yamaguchi M, Komori A, Nakata A, Nishiwaki Y, et al. N-cadherin mediates retinal lamination, maintenance of forebrain compartments and patterning of retinal neurites. Development. 2003;130(11):2479–94. 10.1242/dev.00465 12702661

[51] Malicki J. Cell fate decisions and patterning in the vertebrate retina: the importance of timing, asymmetry, polarity and waves. Curr Opin Neurobiol. 2004;14(1):15–21. 10.1016/j.conb.2004.01.015 15018933

[52] Yu J, Lei K, Zhou M, Craft CM, Xu G, Xu T, et al. KASH protein Syne-2/Nesprin-2 and SUN proteins SUN1/2 mediate nuclear migration during mammalian retinal development. Hum Mol Genet. 2011;20(6):1061–73. 10.1093/hmg/ddq549 21177258 PMC3043658

[53] Turner DL, Cepko CL. A common progenitor for neurons and glia persists in rat retina late in development. Nature. 1987;328(6126):131–6. 10.1038/328131a0 3600789

[54] Karki S, Holzbaur EL. Cytoplasmic dynein and dynactin in cell division and intracellular transport. Curr Opin Cell Biol. 1999;11(1):45–53. 10.1016/s0955-0674(99)80006-4 10047518

[55] Torisawa T, Kimura A. The Generation of Dynein Networks by Multi-Layered Regulation and Their Implication in Cell Division. Front Cell Dev Biol. 2020;8:22. 10.3389/fcell.2020.00022 32083077 PMC7004958

[56] Eschbach J, Dupuis L. Cytoplasmic dynein in neurodegeneration. Pharmacol Ther. 2011;130(3):348–63. 10.1016/j.pharmthera.2011.03.004 21420428

[57] Mattapallil MJ, Wawrousek EF, Chan CC, Zhao H, Roychoudhury J, Ferguson TA, et al. The Rd8 mutation of the Crb1 gene is present in vendor lines of C57BL/6N mice and embryonic stem cells, and confounds ocular induced mutant phenotypes. Invest Ophthalmol Vis Sci. 2012;53(6):2921–7. 10.1167/iovs.12-9662 22447858 PMC3376073

[58] Higginbotham H, Bielas S, Tanaka T, Gleeson JG. Transgenic mouse line with green-fluorescent protein-labeled Centrin 2 allows visualization of the centrosome in living cells. Transgenic Res. 2004;13(2):155–64. 10.1023/b:trag.0000026071.41735.8e 15198203

[59] Zhang H, Hanke-Gogokhia C, Jiang L, Li X, Wang P, Gerstner CD, et al. Mistrafficking of prenylated proteins causes retinitis pigmentosa 2. FASEB J. 2015;29(3):932–42. 10.1096/fj.14-257915 25422369 PMC4422365

[60] Wu CH, Zong Q, Du AL, Zhang W, Yao HC, Yu XQ, et al. Knockdown of Dynamitin in testes significantly decreased male fertility in Drosophila melanogaster. Dev Biol. 2016;420(1):79–89. 10.1016/j.ydbio.2016.10.007 27742209

[61] Hanke-Gogokhia C, Wu Z, Sharif A, Yazigi H, Frederick JM, Baehr W. The guanine nucleotide exchange factor Arf-like protein 13b is essential for assembly of the mouse photoreceptor transition zone and outer segment. J Biol Chem. 2017;292(52):21442–56. 10.1074/jbc.RA117.000141 29089384 PMC5766971

[62] Hanke-Gogokhia C, Chiodo VA, Hauswirth WW, Frederick JM, Baehr W. Rescue of cone function in cone-only Nphp5 knockout mouse model with Leber congenital amaurosis phenotype. Mol Vis. 2018;24:834–46. 30713422 PMC6334983

[63] Ying G, Frederick JM, Baehr W. Deletion of both centrin 2 (CETN2) and CETN3 destabilizes the distal connecting cilium of mouse photoreceptors. J Biol Chem. 2019;294(11):3957–73. 10.1074/jbc.RA118.006371 30647131 PMC6422093

