# Supplementary figures and images for: Effect of conditional deletion of cytoplasmic dynein heavy chain DYNC1H1 on postnatal photoreceptors
Source: PLoS One. 2021 Mar 11;16(3):e0248354. doi: 10.1371/journal.pone.0248354 (PMC7951903; doi:10.1371/journal.pone.0248354)

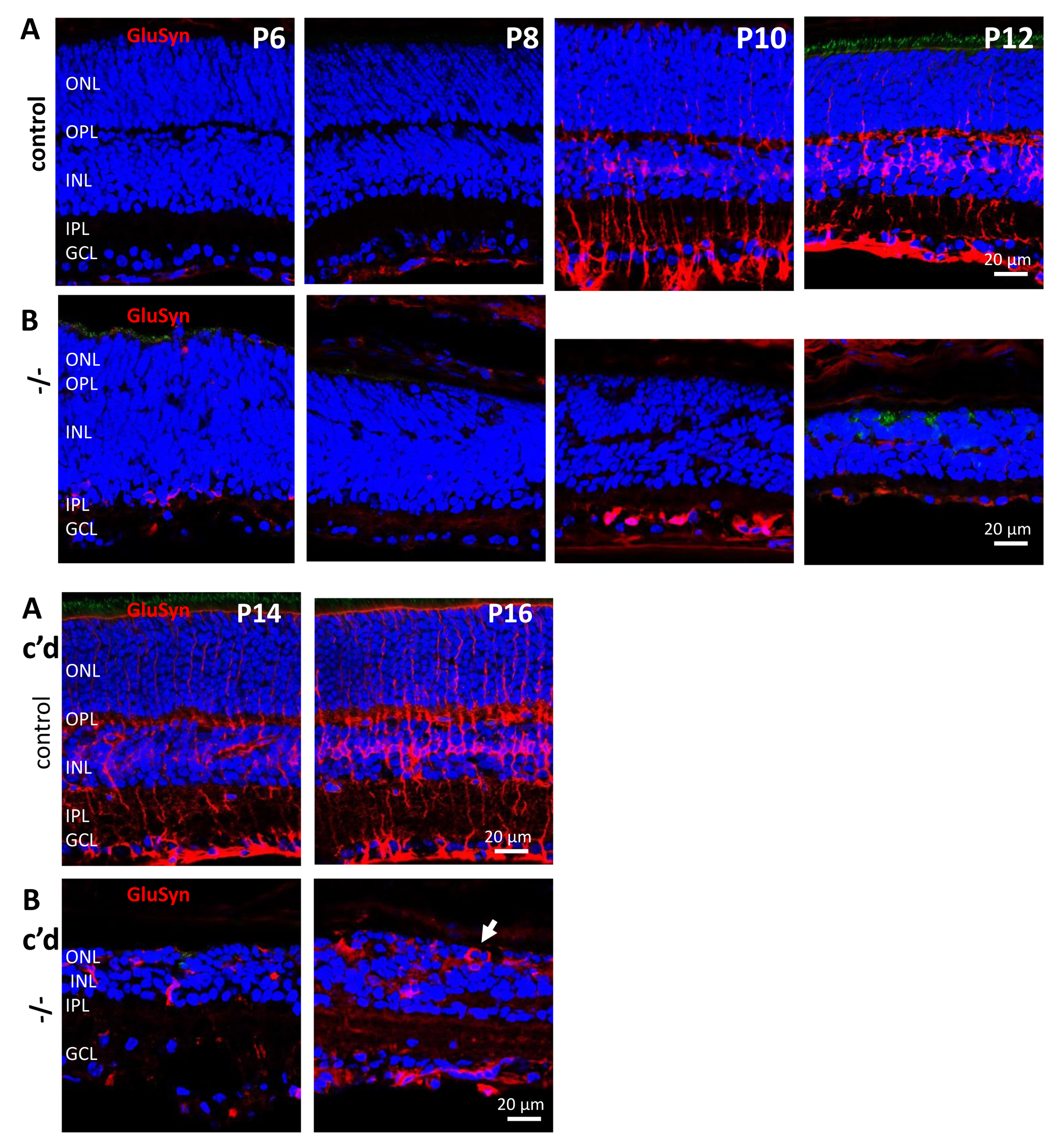

Supplement: S1 Fig — Rows A, B, control sections (row A) and Dync1h1F/F;Six3Cre knockout sections (row B) probed with anti-glutamine synthetase (GluSyn) recognizing Müller Glia with nuclei centered in the INL. Control MGC endfoot structures start to develop at P8 and processes reach their full extension to the OLM by P14. At P16 few GluSyn-positive nuclei are seen in Dync1h1F/F;Six3Cre (arrow in row B). (TIF) [file pone.0248354.s001.tif]

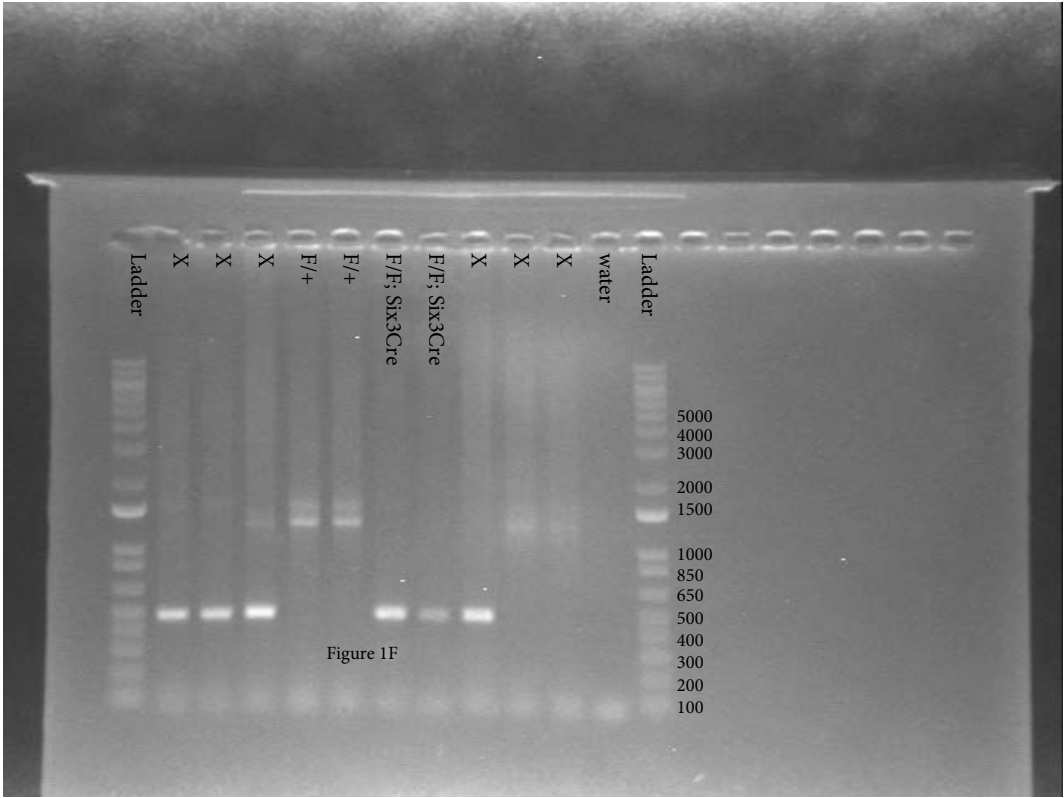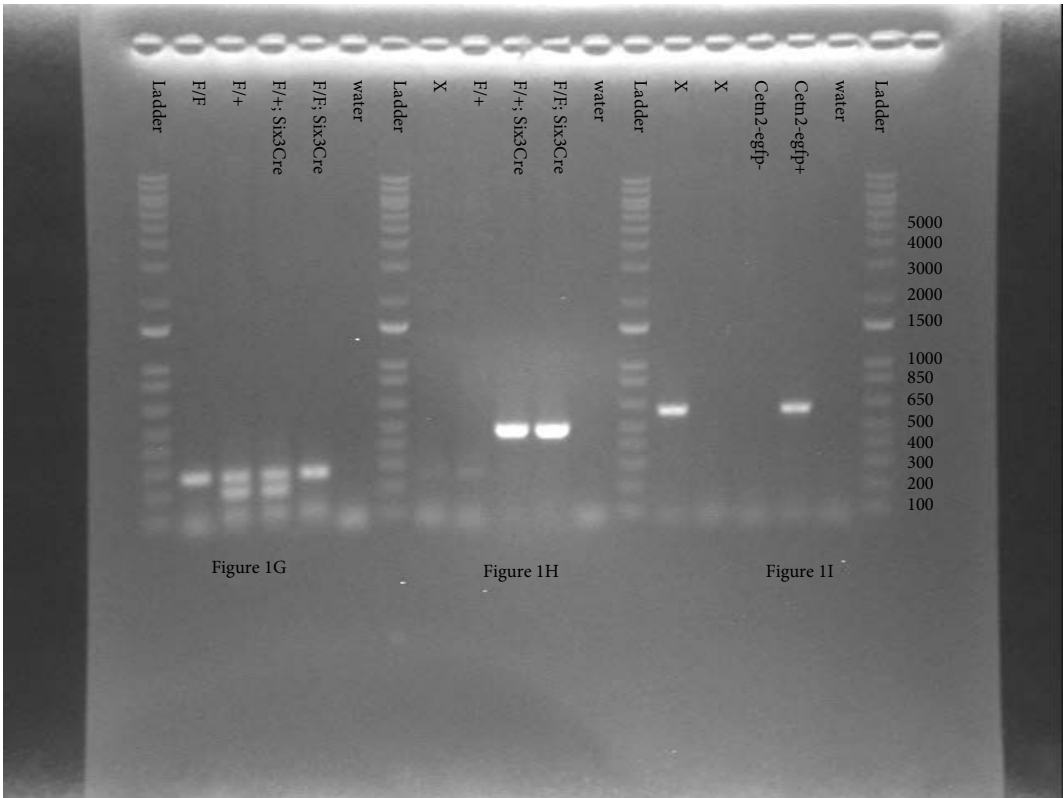

Supplement: S1 Raw images — (PDF) [file pone.0248354.s002.pdf]
